# Held but not healed – Why coercive practices undermine mental health and wellness

**DOI:** 10.1371/journal.pmen.0000594

**Published:** 2026-04-08

**Authors:** Sandra Ferreira

**Affiliations:** Aves Mental Health, Global Network Manager, Johannesburg, South Africa; PLOS: Public Library of Science, UNITED KINGDOM OF GREAT BRITAIN AND NORTHERN IRELAND

## Introduction

### Author positionality and purpose

I write this article as someone who has experienced multiple involuntary practices within mental health care, both in private and public care, predominantly in South Africa but also abroad. My perspective is shaped not only by engagement with research and policy but also by having been subjected to numerous coercive interventions, including but not limited to physical and chemical restraints and involuntary admissions carried out without my consent. That experience informs both my motivation for this work and my understanding of the gaps that persist in dominant clinical and academic narratives about coercion.

With respect to my community, I write this while consciously recognising my own positionality. I write from a position of relational safety and privilege. Through my work as a mental health advocate, I am afforded professional credibility and a degree of psychological safety. I also hold a postgraduate degree and have learned the language of scholarly communications. These forms of privilege shape not only my capacity to analyse coercive practices, but also my ability to speak publicly about them without facing the same levels of risk, dismissal, or retaliation that many others encounter, particularly those who have experienced similar or worse harms but do not have access to these forms of safety or professional legitimacy.

This positionality matters because it exposes an inequitable system of voice, in which legitimacy is unevenly distributed, and those harmed by coercive practices are least likely to be heard or believed.

In writing this piece, I understand my positionality as also carrying ethical responsibility. I endeavour to use the access, safety, and professional legitimacy available to me with care and accountability, not to speak for the community, but to resist the erasure of perspectives that are routinely marginalised. This work is offered as an act of mutuality and solidarity, intended to do justice to the experiences of those whose voices are constrained by systems that continue to privilege certain forms of knowledge while excluding others.

In naming both my experience and my privilege, I do not seek to claim neutrality. Rather, I aim to be transparent about the conditions that make this analysis possible. Lived experience offers access to dimensions of coercion that are not captured by clinical metrics alone, including how these interventions are internalised. To centre only professionally sanctioned knowledge while excluding those most affected is not objectivity, but epistemic and experiential limitation.

The purpose of this article is to specifically illuminate the long-term consequences of coercive practices, attending not only to their immediate effects but to how they are remembered and carried forward over time. In doing so, I seek to move beyond narrow clinical framings of outcome and recovery, and instead foreground the enduring relational, psychological, and existential impacts that are often left unexamined. The recommendations that follow are therefore not offered as abstract ideals, but as grounded responses to lived realities aimed at reducing harm and reorienting systems of care toward approaches that recognise the centrality of dignity and true care for those subjected to coercion.

### How, where, and why coercion is used

Coercive practices remain a routine feature of mental health care systems across many global contexts [1,2]. These practices, including involuntary admission, physical and chemical restraint, seclusion, and community treatment orders, are most commonly employed in acute and crisis settings. Although often framed as time-limited interventions, their effects frequently extend far beyond the moment of systemic intervention. Coercion is typically justified through narratives of risk management and clinical necessity, presented as actions taken in the “best interests” of individuals deemed unable to make decisions for themselves.

Within dominant psychiatric frameworks, coercion is positioned as an unfortunate but necessary response to perceived danger to the individual, to others, or to public order. Concepts such as lack of insight, impaired capacity, and non-compliance are routinely mobilised to legitimise the suspension of autonomy, consent, and human rights. In these moments, control is prioritised over understanding and empathy, and containment is framed as care.

Yet this framing often obscures a critical question: who are these coercive practices really safeguarding, and is it truly about care?

Coercive interventions are most often enacted at points of heightened vulnerability, during psychosis, severe distress, suicidality, or emotional overwhelm, when people are least able to advocate for themselves or challenge institutional authority. The power imbalance in these encounters is profound. Once coercion is initiated, the person subjected to it is rarely treated as a credible narrator of their own experience; distress becomes evidence of pathology, and resistance further justification for force.

In addition, the persistence of coercive practices is often explained in terms of necessity rather than effectiveness. They are described as last resorts, despite evidence that many individuals experience them as predictable rather than exceptional [3]. Institutional pressures, including under-resourced services, risk-averse cultures, and legal frameworks that prioritise liability over relational care, play a central role in normalising coercion. In such contexts, rapid control becomes a proxy for “good care,” even when long-term outcomes are harmful.

Critically, the rationale for coercion is rarely examined from the perspective of those who endure it. While policies and clinical guidelines emphasise presumed intent, such as protection or stabilisation, they seldom account for impact. The emotional, psychological, and relational consequences of coercive practices are frequently minimised or reframed as unavoidable side effects rather than harms in their own right. This gap between intention and lived experience reflects a broader epistemic hierarchy within mental health systems, where clinical authority is privileged over experiential, living accounts. As a result, coercion continues to be implemented and defended without sufficient interrogation of whether it supports long-term wellness or the needs defined by those most affected.

This article argues that understanding coercion solely through institutional and clinical logics is insufficient. To meaningfully evaluate its role and impact, lived experience must be centred not as anecdote, but as a legitimate and necessary epistemology. In practice, this requires systematically embedding first-person accounts into research design, service evaluation, and policy development, through co-produced methodologies and decision-making processes that give equal weight to experiential knowledge alongside clinical evidence.

Only by examining how coercion is experienced and carried forward in people’s lives can its true effects on mental health and wellbeing be understood.

### Lived experience epistemology against diagnostic dominance

Epistemic authority in mental health has historically been concentrated in clinicians, researchers, and institutional diagnostic systems, often privileging standardized assessments and procedural frameworks over testimony and lived experience [4].

The Diagnostic and Statistical Manual of Mental Disorders (DSM) is the American Psychiatric Association’s primary classification system for diagnosing mental health conditions and is widely used internationally in clinical practice, research, policy, and insurance contexts. Ongoing discussions surrounding DSM-6 have renewed critique of how psychiatric classification continues to prioritise biomedical and categorical explanations of distress while marginalising social, relational, and experiential dimensions [5,6]. Diagnostic categories do not merely describe distress; they shape clinical reasoning, research agendas, funding priorities, and legal thresholds for coercive intervention.

In many jurisdictions, diagnosis functions as a gateway to coercion, providing clinical and legal legitimacy for involuntary treatment. Against this backdrop, lived experience - knowledge generated through direct, embodied engagement with mental health systems - emerges not merely as valid but as essential for understanding, challenging, and transforming these structures.

Critiques of psychiatric diagnostic systems have long noted that classification frameworks such as the DSM reflect cultural assumptions, institutional priorities, and normative constructions of distress rather than neutral universal truths about the human and living experience. Academics argue that DSM criteria can homogenise diverse experiences by decontextualising distress and limiting attention to sociocultural context, thereby reinforcing dominant perspectives [7,8].

In contrast, lived experience foregrounds impact, ethical consequence, and the internalised meaning of interventions rather than adherence to categories or procedures. Across research agendas, funding decisions, and individual care, despite its depth and insight, lived experience is often tokenised or dismissed, while diagnostic knowledge is presumed authoritative. All knowledge is situated, practised, and lived. Without epistemic authority, lived experience risks being extracted for institutional ends rather than genuinely recognised, reinforcing the hierarchies it is intended to challenge [9].

The use of language that lived experience is a valid epistemology is deliberate. It reflects an attempt to do more than simply call for greater attentiveness to lived experience; it seeks to unsettle the assumptions that determine what is recognised as valid knowledge within the field. While such terminology may feel unfamiliar or even obscure, this friction is, in part, the point: it signals a shift from inclusion as consultation toward inclusion as equal partnership.

Lived experience provides access to dimensions of care often overlooked by standard measures. Individuals subjected to coercive practices carry an understanding of fear, powerlessness, trauma, and relational rupture - insights central to understanding well-being and long-term outcomes [10]. The marginalisation of lived experience constitutes epistemic injustice, especially in contexts of coercion, where credibility is routinely undermined precisely because of the experiences individuals seek to articulate [11].

In response, lived experience movements have asserted their role as knowledge producers. International guidance from the World Health Organisation (WHO) and the United Nations (UN) -aligned frameworks emphasize the meaningful involvement of people with lived experience across research, policy, and service design. Organisations such as aves Mental Health demonstrate how co-

produced qualitative research positions experiential knowledge as foundational rather than supplementary and that involving people with lived experience in leadership and policy development generates knowledge that is ethically and conceptually distinct [12,13]. Recognising lived experience as a valid epistemology does not reject clinical knowledge or research expertise; it rebalances whose knowledge is legitimised, supported, and afforded authority.

In simple terms, centring lived experience should not become another layer of abstraction, but a clear commitment to placing lived experience perspectives at the heart of how coercion is understood, evaluated, and ultimately reformed.

### Coercion through lived experience: Impacts on recovery

Before engaging further with the concept of recovery, it is important to clarify my position. I do not personally find the term recovery fully reflective of my experiences. For clarity and alignment with dominant mental health discourse, I use the term throughout this article, but this should not be read as an uncritical endorsement.

My experiences of distress, crisis, and coercive intervention have not been discrete episodes from which one simply “recovers.” They have been formative, shaping relationships, values, and my sense of self. I therefore speak more readily of wellness or wellbeing or philosophically, of *becoming* or *being* as an ongoing, non-linear process of living and meaning-making, rather than an endpoint defined by the absence of symptoms or functional compliance.

This distinction matters because much of the evidence used to justify coercive practices relies on a narrow, clinically defined understanding of recovery that prioritises stability and symptom reduction over dignity, agency, and self-defined wellbeing. Viewed through this lens, the limitations of the evidence base become apparent.

Across my own experiences of involuntary admission and those shared by peers, I have yet to encounter anyone who describes coercion as a catalyst for sustained wellness. What is consistently reported instead are experiences of fear, humiliation, physical force, and erosion of dignity.

In moments of acute distress, individuals are frequently reduced to being risks that must be managed. Being restrained, transported by police, or confined without explanation perpetuates the dehumanisation of other similar carceral processes. Information about legal status, timeframes, or rights is often absent or rendered invisible, never mind thoughts and preferences about treatment. Although people with mental health conditions are often perceived as dangerous, evidence consistently shows they are more likely to be victims of crime than perpetrators [14].

Clinical explanations for repeated hospitalisation or “relapse” often focus on individual non-adherence, such as medication refusal, self-medicating, or disengagement from services. While these factors may contribute, such explanations overlook the harms inflicted by coercive care itself. The psychological impact of being overpowered, stripped of autonomy, and treated as incapable is rarely considered a clinically relevant variable.

From lived experience, coercion does not function as a neutral interruption of crisis. It leaves lasting imprints. It teaches people that distress will be met with force, that honesty invites punishment, and that survival within the system depends on concealment rather than engagement. These lessons are incompatible with *being* well. They undermine trust and fracture an individual’s sense of agency at precisely the point where agency is most needed.

### Who decides “best interests” when rights are ignored?

Coercive practices are rarely framed as punitive; instead, they are legitimised through claims of protection and necessity. Once autonomy and consent are suspended, “best interests” becomes the primary moral framework through which rights violations are justified.

Within dominant mental health systems, best interests are typically determined by clinicians or legal bodies based on assessments of risk, capacity, and compliance. These determinations are presented as objective, yet they are shaped by institutional priorities, risk-averse cultures, and socially constructed assumptions about mental distress. The person subjected to coercion is rarely recognised as an authority on their own needs or values once this threshold is crossed.

This dynamic raises a fundamental human rights concern. When best interests are defined externally, they displace the individual’s understanding of safety, dignity, and recovery. The result is substituted decision-making that prioritises control over relational care.

International human rights law, particularly the UN Convention on the Rights of Persons with Disabilities (UNCRPD), directly challenges best-interests-based overrides in mental health care [15]. Article 12 affirms equal recognition before the law, while the Committee explicitly rejects regimes that allow others to decide what is in a person’s best interests based on perceived incapacity or psychosocial disability. Under the UNCRPD, the appropriate standard is respect for *rights, will, and preferences,* not best interests.

A will-and-preferences approach asks not whether professionals believe an intervention is necessary, but how support can be mobilised to enable choice, communication, and consent. It requires attention to advance directives, crisis plans, trusted supporters, and the relational context in which decisions are made. Crucially, it also demands tolerance of uncertainty, recognising that eliminating all risk is neither possible nor ethically justifiable when it comes at the expense of dignity and agency. *A rights-based emphasis on will and preferences makes power visible*. It clarifies who decides, on what basis, and with what consequences.

The displacement of will and preferences is reinforced through mental health legislation across many jurisdictions, where subjective thresholds for coercion are embedded in law. In numerous countries, involuntary care is authorised based on a clinician’s belief or “reasonable grounds” to assume risk, granting wide discretionary power with limited procedural safeguards. Such reliance on subjective judgment is ethically indefensible.

Even in circumstances where coercion is deemed necessary to prevent immediate harm, an individual’s rights, will, and preferences are often not only temporarily overridden but continue to be marginalised within longer-term care. What may begin as an acute, time-limited intervention can extend into ongoing practices in which decision-making remains predominantly clinician-led, with insufficient attention to restoring the person’s agency once the crisis has passed. In this way, the justification of necessity risks expanding beyond its original scope, normalising a sustained diminishment of autonomy rather than a careful, time-bound response to risk. A more ethically coherent approach would require that any initial suspension of will and preference be explicitly limited, continually reviewed, and actively reversed.

If mental health systems are genuinely committed to care and wellbeing, practices that systematically override will and preferences must be critically examined rather than defended through paternalistic language.

### Evidence fails to support coercion in mental health

Coercive practices are frequently defended as reducing immediate risk, preventing harm, or stabilising acute crises. Research has largely focused on short-term outcomes such as containment, symptom reduction, or incident prevention within institutional settings [16]. These metrics reflect organisational priorities rather than wellbeing or sustained community participation. Notably absent is robust evidence that coercive interventions improve long-term quality of life, autonomy, relational functioning, or personal well-being. Longitudinal studies show coercion is more often associated with disengagement from services, avoidance of future care, and heightened distress [17].

It is, however, important to acknowledge that, while some individuals report that coercive interventions were helpful in their experience, these accounts are far from universal. Importantly, these cases that describe coercion as beneficial occurred before the adoption of the United Nations Convention on the Rights of Persons with Disabilities (UNCRPD) in 2006, in a context where legal and human rights frameworks did not fully recognise the agency and autonomy of people with psychosocial disabilities. Including this caveat situates these experiences historically and underscores that, although some lived experiences of coercion may be positive, they exist alongside a broader and more contested landscape in which coercion is frequently experienced as harmful, disempowering, or traumatic.

The continued reliance on coercive practices in the absence of demonstrated recovery benefits raises an important question*: why do these interventions continue to persist, often becoming the default approach?* Part of the answer lies in structural apathy. Coercion is embedded in legal frameworks, professional training, and risk-averse institutional cultures that reward immediate control over long-term outcomes. As an example, first responders who are often police services interpret distress, refusal, or emotional escalation as indicators of risk rather than as communicative acts or responses to context. With this, de-escalation is frequently positioned as a precursor to restraint rather than as a relational alternative to coercive confinement.

### Cognitive institutionalisation: The coercive power of psychiatric language

Coercion in mental health care is not enacted through force alone. It is also produced and sustained through language and systemic indoctrination - through the words used to describe people, interpret their actions, and legitimise interventions. Psychiatric language does not merely reflect clinical reality; it actively shapes it, determining who is seen as credible, who is authorised to decide, and whose perspective can be ignored. I describe this process as **cognitive institutionalization**: the internalisation of institutional logics, categories, and hierarchies through language and thought, shaping how people are perceived and ultimately treated.

Terms such as “non-compliant,” “lacks insight,” or “treatment-resistant” are often presented as neutral descriptors but carry implicit moral judgments. They frame disagreement as pathology and refusal as a deficit. Once applied, such language narrows interpretive possibilities and renders coercion reasonable or inevitable.

Through cognitive institutionalisation, people are positioned as objects of management rather than participants in their own care. Diagnostic labels further entrench this dynamic, often rendering personal accounts unreliable, especially during a crisis. Decisions are made about people rather than with them, and clinical discussions proceed in ways that exclude the individual’s voice, even when they are physically present. Cognitive institutionalisation has a direct and material impact on both internalised stigma and the reproduction of structural stigma, shaping how individuals understand themselves and how systems justify unequal treatment.

From lived experience, the consequences of these processes are palpable. During one of my admissions, I was administered medication that I knew would rapidly sedate me. Having experienced similar interventions without consent various times in the past, I became visibly distressed. What mattered in that moment was not the intervention itself, but how it was interpreted and enacted. Rather than reading my distress as resistance or non-compliance, my clinician recognised it as fear rooted in prior trauma. She paused, made eye contact, and acknowledged my distress. That brief act of relational presence did not remove the coercive context or dissolve the power imbalance, but it restored my sense of personhood. I was treated not as a problem to be managed, but as a human being experiencing fear, and the impact of that interaction has endured to this day.

An especially revealing expression of cognitive institutionalization is the routine prioritisation of the term *“patient”* over *“person.”* While not inherently harmful, the term patient carries institutional and hierarchical connotations: passivity, dependency, and an expectation of compliance. Its default use subtly repositions individuals as recipients of intervention rather than as people with intersectional identities, histories, and values beyond the clinical encounter.

Language and process are not merely descriptive; it is constitutive. Shifting toward person-led language and processes is therefore not about sensitivity alone, but about actively resisting cognitive institutionalisation. Language can either entrench coercion or create space for true wellness. Dismantling coercive practice requires dismantling coercive ways of thinking and being.

## Recommendations

### Legally embed advance directives and eliminate substituted decision-making

Reducing coercion requires more than policy statements; it demands a fundamental transformation of mental health systems, both structurally and relationally. Advance directives and crisis plans must be legally recognised, routinely supported, and meaningfully implemented, ensuring that individuals’ will and preferences guide care before crises arise and reducing and ultimately eradicating reliance on substituted decision-making [18,19].

### Shift care from institutions to community-led supports

Care must shift decisively from acute, institutional settings to adequately resourced, community-based, but ideally community-led supports. Evidence demonstrates that peer-led services, psychosocial supports, housing-first approaches, and culturally responsive care improve engagement, prevent crisis escalation, and lower reliance on coercion [20]. This shift must be accompanied by sustained community education so that families and communities are equipped to understand rights, challenge coercive norms, and support non-institutional responses to distress, rather than defaulting to institutional authority.

### Operationalise human rights frameworks in everyday practice

Human rights frameworks, particularly the UN Convention on the Rights of Persons with Disabilities, must be embedded in clinical education, legislation, and practice, serving not as abstract ideals but as operational guides for everyday decisions [15].

### Disrupt coercion through reflective practice and cognitive de-institutionalisation

Professionals must interrogate their own cognitive institutionalisation - the assumptions, language, and risk-focused thinking that reproduce coercion not only through formal orders but through everyday interactions with people in care. While systemic policy and legislative changes can take years to implement, reflective practice and critical self-awareness can have an immediate and profound impact. By consciously examining how to interpret distress, communicate with those experiencing crises, and make routine decisions, clinicians can reduce coercive dynamics and create space for relational, rights-respecting care even within existing institutional structures. These micro-

level shifts, though often overlooked, are essential levers for change and can transform the lived experience of care long before formal reforms are realised.

This responsibility extends beyond individual clinicians to medical and mental health education, as well as to publishers and media, who must adopt language and narratives that resist normalising coercion and promote rights-based language and approaches.

### Embed lived experience as a core epistemology

Finally, lived experience must be recognised as a core epistemic resource, with power genuinely shared in research, policy, leadership, and service design [13]. Upcoming revisions to diagnostic frameworks, including DSM-6, represent a pivotal moment to confront how psychiatric classification has historically legitimised coercion. Integrating lived experience at the core of review is not optional; it is essential to challenge entrenched hierarchies, disrupt the concept of risk-based control, and ensure that diagnostic criteria serve human dignity rather than institutional convenience.

## Concluding remarks

Reducing coercion does not mean abandoning care in moments of crisis. It demands a fundamental transformation of where care occurs, how it is delivered, and who holds responsibility for it. Without a shift toward community-led, rights-respecting, and relational systems, people will continue to be held, but true healing and wellness will remain out of reach. The familiar adage that insanity is doing the same thing repeatedly and expecting different results has long been misapplied to those in care. This article demonstrates that the insanity lies not within individuals but within the system itself.

## References

[1] World Health Organization. Guidelines on mental health, human rights and legislation. WHO; 2019.

[2] Pūras D. Report of the Special Rapporteur on the right of everyone to the enjoyment of the highest attainable standard of physical and mental health. United Nations; 2017.

[3] Newton-Howes G, Stanley J. Prevalence of perceived coercion among psychiatric patients: literature review and meta-regression modelling. Psychiatrist. 2012;36(9):335–40. doi: 10.1192/pb.bp.111.037358

[4] Carel H, Kidd IJ. Epistemic injustice in healthcare: a philosophial analysis. Med Health Care Philos. 2014;17(4):529–40. doi: 10.1007/s11019-014-9560-2 24740808

[5] American Psychiatric Association. DSM-5-TR: Diagnostic and Statistical Manual of Mental Disorders. 5th ed. Washington (DC): APA; 2022.

[6] Paris J. The ideology behind DSM-5. J Nerv Ment Dis. 2015;203(9):664–8.26252825

[7] Bredström A. Culture and context in mental health diagnosing: scrutinizing the DSM-5 revision. J Med Humanit. 2019;40(3):347–63. doi: 10.1007/s10912-017-9501-1 29282590 PMC6677698

[8] Kapadia M, Desai M, Parikh R. Fractures in the framework: limitations of classification systems in psychiatry. Front Psychiatry. 2020;11:529. 32699502 10.31887/DCNS.2020.22.1/rparikh PMC7365290

[9] Sharma P. Reparations not remuneration: redefining the future of lived experience. PLOS Ment Health. 2025;2(11):e0000495. doi: 10.1371/journal.pmen.0000495 41662111 PMC12798572

[10] Faulkner A, Basset T. Lived experience and co-production in mental health research. J Ment Health. 2020;29:1–10.31985302

[11] Kidd IJ, Carel H. Epistemic Injustice in Mental Health. J Med Ethics. 2023;49:456–62.

[12] Global Mental Health Peer Network. Co-production and Lived Experience in Global Mental Health Research. GMHPN Report; 2024.

[13] Sunkel C, Sartor C. Lived experience in leadership and Policy. Int J Ment Health Syst. 2022;16:54.36424651

[14] Teplin LA, McClelland GM, Abram KM, Weiner DA. Crime victimization in adults with severe mental illness. Arch Gen Psychiatry. 2005;62(8):911. doi: 10.1001/archpsyc.62.8.911 16061769 PMC1389236

[15] United Nations. Convention on the Rights of Persons with Disabilities. UN; 2006. 10.1515/9783110208856.203 18348362

[16] Kalisova L, Priebe S. Short-term outcomes of coercive psychiatric interventions: a systematic review. BMC Psychiatry. 2018;18:289.30195335

[17] Kisely S, Campbell LA, O’Reilly R. Compulsory community and inpatient treatment for people with severe mental disorders. Cochrane Database Syst Rev. 2017;2017(3):CD004408. 10.1002/14651858.CD004408.pub5 PMC6464695 28303578

[18] Campbell LA, Kisely S. Advance directives and coercion reduction. Psychiatric Serv. 2017;68:1063–70.

[19] aves Mental Health. Advanced directive for mental health care. Available from: https://www.aves-mh.org/resources

[20] Thornicroft G, Deb T, Henderson C. Community mental health care worldwide: lessons from global research. Lancet Psychiatry. 2016;3:75–87. 10.1002/wps.20349 PMC5032514 27717265

